# Editorial: Reproductive biotechnologies and challenges in their application, volume II

**DOI:** 10.3389/fvets.2026.1890624

**Published:** 2026-07-01

**Authors:** Ştefan G. Ciornei, Graça Lopes, Mihai Cenariu

**Affiliations:** 1 Faculty of Veterinary Medicine, “Ion Ionescu de la Brad” University of Life Sciences Iasi, Iaşi, Romania; 2 School of Medicine and Biomedical Sciences (ICBAS), University of Porto, Porto, Portugal; 3 Faculty of Veterinary Medicine, University of Agricultural Sciences and Veterinary Medicine of Cluj-Napoca, Cluj-Napoca, Romania

**Keywords:** biomarker analysis, embryo-stage optimization, fertility assessment, hormonal synchronization, oxidative stress, proteomics, reproductive diagnostics, semen preservation

The Research Topic “*Reproductive biotechnologies and challenges in their application, volume II”* published in Frontiers in Veterinary Science (*Animal Reproduction—Theriogenology*) presents a broad overview of current advances in animal reproductive biotechnology and the practical challenges associated with fertility management, semen preservation, embryo transfer, hormonal regulation, and reproductive welfare. Together, the 13 articles emphasize the growing importance of biotechnology in improving reproductive efficiency, livestock productivity, and animal health, while also showing the biological and ethical limitations that continue to constrain the field.

## 1 Male fertility studies

### 1.1 Semen preservation and quality

Several studies focused on semen preservation and sperm quality, which represent central concerns in reproductive biotechnology. Khalil et al. investigated rabbit semen cryopreservation using different cryoprotectants in a Tris-based extender. Their study demonstrated that optimized cryoprotectant combinations including 4% DMSO enriched with either 50 mM trehalose or 50 mM sucrose significantly improved sperm motility, viability, and post-thaw fertility potential. This research contributes to the ongoing effort to minimize cryodamage during freezing, one of the main limitations of semen preservation technologies (1). Similarly, Alfattah explored the use of europium oxide nanoparticles (EONPs) during chilled rabbit semen storage. The nanoparticles enhanced antioxidant activity and mitochondrial function, thereby improving semen quality and extending preservation time. The findings suggest that nanotechnology may become an innovative strategy for reducing oxidative stress during semen storage. Another semen-related investigation by Song et al. evaluated the effects of chitosan supplementation during low-temperature buck semen storage. Chitosan improved sperm quality and positively influenced seminal plasma metabolites, indicating that biochemical additives may protect spermatozoa against cold-induced damage.

### 1.2 Sperm biology and fertility biomarkers

Singh et al. analyzed the seasonal effects on boar semen fertility in subtropical climates. They demonstrated that summer heat stress negatively affected mitochondrial membrane potential and antioxidant biomarkers, ultimately reducing fertility. Their work highlights the strong relationship between environmental stressors and reproductive efficiency (2), emphasizing the importance of climate-adapted reproductive management strategies.

Bodu et al. investigated sperm cellular and nuclear dynamics associated with ram fertility. Their study identified structural sperm characteristics closely associated with reproductive success (such as sperm nuclear shape assessed using Fourier harmonic amplitude analysis), thus supporting growing evidence that advanced sperm function testing provides greater predictive accuracy for fertility outcomes compared to conventional semen analysis (3). Likewise, Sevilla et al. examined the relationship between age, scrotal circumference, and sperm morphology in Brahman bulls using a modified fixation technique. They found that age and testicular development significantly influenced sperm quality, reinforcing the importance of andrological evaluation in breeding programs (4).

Proteomic and metabolomic approaches emerged as modern tools for reproductive assessment and are increasingly integrated into reproductive biotechnology (5). Torres Hualla et al. performed proteomic and functional analyses of alpaca sperm after *in vitro* capacitation with follicular and oviductal fluids. Their findings demonstrated that reproductive fluids modify sperm protein expression and functionality, improving the understanding of fertilization mechanisms in camelids. This research may support future assisted reproductive technologies in species with historically limited reproductive success.

## 2 Female fertility studies

### 2.1 Embryo production and reproductive management

Embryo transfer and ovulation management represented another major research direction. Ciornei reported on direct embryo transfer in sheep and observed that hatched blastocysts increased conception rates compared with conventional approaches. The study supports the assessment and optimization of embryo developmental stages prior to transfer as a practical strategy for improving reproductive outcomes in sheep production systems (6). In another study involving synchronized ewes, Cox et al. evaluated phase-specific GnRH administration and its effects on ovarian function, ovulation timing, and fertility. Their results showed that targeted hormonal protocols improved synchronization efficiency and fertility outcomes, demonstrating the importance of precise endocrine control in reproductive management (7).

### 2.2 Reproductive health and welfare

Beyond fertility enhancement, some studies addressed reproductive pathology and animal welfare. A case report by Ciornei et al. described unilateral uterine torsion in a non-pregnant Siberian Husky, providing clinical insights into diagnosis, treatment, and reproductive implications. Although uncommon, the condition highlighted the importance of rapid clinical intervention and careful reproductive monitoring in companion animals. Meanwhile, Van den Branden et al. investigated pain and stress responses in mares undergoing transvaginal ultrasound-guided follicular aspiration. By evaluating physiological and behavioral parameters, the authors demonstrated that reproductive procedures can significantly influence animal welfare. Their findings emphasize the need for improved analgesic protocols and welfare-conscious reproductive practices in veterinary medicine (8).

Wang et al. provided a comprehensive review that highlights the major infectious and non-infectious causes of reproductive disorders in sows and emphasizes integrated prevention and “One Health” management strategies to improve swine reproductive health and productivity.

### 2.3 Study models

A particularly innovative contribution involved the establishment of an immortalized bovine luteal cell line by Fei et al. The researchers successfully developed a stable cellular model for studying luteal physiology and hormone production. This advancement provides a valuable experimental tool for investigating corpus luteum function, reproductive endocrinology, and fertility regulation in cattle.

## 3 Scientific trends and future directions

Collectively, the articles reveal several common scientific trends. First, oxidative stress appears repeatedly as a major factor impairing sperm survival and fertility. Several authors explored antioxidant-based interventions, including nanoparticles, chitosan supplementation, and optimized extenders, to improve semen preservation. Second, the studies emphasize precision reproductive management through hormonal synchronization, advanced sperm evaluation, and embryo-stage optimization. Third, molecular technologies such as proteomics and biomarker analysis are shown to increasingly shape the future of fertility assessment and reproductive diagnostics.

The Research Topic also illustrates the diversity of species benefiting from reproductive biotechnology research, including rabbits, sheep, goats, cattle, boars, alpacas, horses, and dogs. This diversity reflects the broad applicability of reproductive technologies across livestock production, companion animal medicine, and potentially wildlife conservation. However, the studies also demonstrate that species-specific physiological differences remain a major challenge, requiring tailored reproductive protocols rather than universal approaches.

Another significant aspect refers to the balance between productivity and animal welfare. While reproductive biotechnologies aim to improve fertility and breeding efficiency, stress, pain, inflammation, as well as ethical considerations were shown to be associated with intensive reproductive management (9). Such concerns reinforce the importance of developing technologies that are not only effective but also humane and ethically responsible.

Overall, the articles demonstrate that animal reproductive biotechnology is rapidly evolving into a multidisciplinary and precision-oriented field. Advances in cryobiology, endocrinology, proteomics, and assisted reproductive technologies are improving fertility outcomes and reproductive efficiency across species. Persistent challenges, including oxidative stress, environmental influences, and welfare concerns, continue to shape ongoing research efforts. The Research Topic ultimately highlights the transition of reproductive biotechnology from traditional reproductive management toward integrative, molecular, and welfare-conscious approaches that may significantly influence the future of veterinary reproductive medicine and sustainable animal production.
